# Supplementary material for: Time-series transcriptome analysis identified differentially expressed genes in broiler chicken infected with mixed Eimeria species
Source: Front Genet. 2022 Aug 8;13:886781. doi: 10.3389/fgene.2022.886781 (PMC9393255; doi:10.3389/fgene.2022.886781)
Supplement: Supplementary file 2 [file DataSheet1.ZIP › 7dpi_GO.Gsea.1625106495923/GOBP_EMBRYONIC_CAMERA_TYPE_EYE_FORMATION.html]

Details for gene set GOBP\_EMBRYONIC\_CAMERA\_TYPE\_EYE\_FORMATION[GSEA]

|  || Dataset | TMM\_7dpi\_gct\_format\_7dpi\_gct\_format.Class\_4dpi.cls #PC\_versus\_NC.Class\_4dpi.cls #PC\_versus\_NC\_repos |
| Phenotype | Class\_4dpi.cls#PC\_versus\_NC\_repos |
| Upregulated in class | 0 |
| GeneSet | GOBP\_EMBRYONIC\_CAMERA\_TYPE\_EYE\_FORMATION |
| Enrichment Score (ES) | -0.94496787 |
| Normalized Enrichment Score (NES) | -1.8159356 |
| Nominal p-value | 0.0 |
| FDR q-value | 0.10966481 |
| FWER p-Value | 0.9222 |
Table: GSEA Results Summary

  

Fig 1: Enrichment plot: GOBP\_EMBRYONIC\_CAMERA\_TYPE\_EYE\_FORMATION      
 Profile of the Running ES Score & Positions of GeneSet Members on the Rank Ordered List

  

| SYMBOL | TITLE | RANK IN GENE LIST | RANK METRIC SCORE | RUNNING ES | CORE ENRICHMENT || 1 | WNT5A | na | 2943 | 0.133 | -0.2158 | No |
| 2 | TWIST1 | na | 3982 | 0.007 | -0.3008 | No |
| 3 | FRS2 | na | 11717 | -1.256 | -0.6691 | Yes |
| 4 | PROX1 | na | 11863 | -1.432 | -0.3666 | Yes |
| 5 | ALDH1A3 | na | 11961 | -1.725 | 0.0041 | Yes |
Table: GSEA details [plain text format]

  

Fig 2: GOBP\_EMBRYONIC\_CAMERA\_TYPE\_EYE\_FORMATION      
 Blue-Pink O' Gram in the Space of the Analyzed GeneSet

  

Fig 3: GOBP\_EMBRYONIC\_CAMERA\_TYPE\_EYE\_FORMATION: Random ES distribution      
 Gene set null distribution of ES for **GOBP\_EMBRYONIC\_CAMERA\_TYPE\_EYE\_FORMATION**
